# Effects of Different Elicitors on Micropropagation, Biomass and Secondary Metabolite Production of *Stevia rebaudiana* Bertoni—A Review

**DOI:** 10.3390/plants12010153

**Published:** 2022-12-29

**Authors:** Kamelia Miladinova-Georgieva, Maria Geneva, Ira Stancheva, Maria Petrova, Mariana Sichanova, Elisaveta Kirova

**Affiliations:** Institute of Plant Physiology and Genetics—Bulgarian Academy of Sciences, Acad. G. Bonchev Street, Building 21, 1113 Sofia, Bulgaria

**Keywords:** antioxidant activity, *in vitro*, nanoparticles, plant cell culture, plant growth regulators, stevioside

## Abstract

*Stevia rebaudiana* Bertoni is a valuable plant whose products are increasingly used in medicine, pharmacy and the food industry. This necessitates the use of biotechnological approaches for its mass propagation. Establishing optimal conditions for *in vitro* cultivation is essential for obtaining high biomass and secondary metabolites production. A large number of articles considering the role of plant growth regulators and other additives in the culture medium in the growth and development of *Stevia* are available in the literature. However, there are no summarized data about the use of nanoparticles in *Stevia* tissue cultures. Therefore, this review also includes the research conducted so far on the effect of nanoparticles on *Stevia* micropropagation. Furthermore, the influence of different elicitors on secondary metabolite production and antioxidant activity of *in vitro*-cultivated *Stevia* plants have been discussed. By referring to the collected literature, we concluded that biotechnological approaches applied to *S. rebaudiana* cultivation might improve the agronomic traits of plants and steviol glycosides production.

## 1. *Stevia rebaudiana* (Bertoni)

### 1.1. Botanical Description 

*Stevia rebaudiana* Bertoni is a herbaceous perennial plant of the Asteraceae family [1]. It is native to South America, in particular Brazil and Paraguay [2]. The plant is approximately 60–75 cm tall, the leaf is sessile and oppositely arranged, the flower is white and the seed is very small [3]. Dr Moises Santiago Bertoni first reported this plant in 1887 [3] when he learned of its unique properties from the Paraguayan, Indians and Mestizos [4]. 

### 1.2. Applications

Stevia is a natural sweetener plant known as Sweet leaf, Sweet herbs and Honey leaf [5,6,7]. The sweetness is due to the presence of more than 30 different steviol glycosides (SGs) found mainly in the leaves [2,8]. SGs are diterpenoids whose chemical structure is based on an aglycone core known as steviol (ent-13-hydroxyur-16-en-19-oic acid) to which a different number and types of sugar molecules are attached [9]. The four abundant SGs are stevioside, rebaudioside A, rebaudioside C and dulcoside A. The content of sweet components varies between 4 and 20% in the dry leaves [10]. Pure steviosides are non-caloric and 300 times sweeter than sucrose or cane sugar [3,11,12]. 

Stevia is used in many forms such as fresh and dried leaves, leaf powder, extracts and liquid concentrates [2]. The powdered form of the leaves has hypoglycemic and body-weight-reducing efficiency [13]. It has been recommended for diabetic and diet-conscious patients [3,13,14]. Stevia has been used in a wide range of processed foods as a substitute for conventional sugars, or artificial dietetics [15]. It is approved by the Food and Drug Administration (FDA) as a dietary supplement [14]. The plant is also a valuable source of proteins, carbohydrates, dietary fibers, vitamins, minerals, essential amino acids, fatty acids, and other health-favorable bioactive compounds such as non-glycosidic labdane diterpenes, flavonoids, phenolic compounds, phytosterols, chlorogenic acids and triterpenes [2,16]. Stevia extract possesses various biological activities, including antihypertensive, antidiabetic, antiobesity, antitumor, anticaries, antioxidant, anti-inflammatory, antimicrobial and antifungal effects and is known to improve kidney function [2,17,18,19,20]. Nowadays, the plant is cultivated in China, Taiwan, Thailand, Korea, Japan, India, Malaysia, Philippines, Hawaii and South America for food and pharmaceutical products [7,17,21,22].

## 2. Propagation of *Stevia rebaudiana* (Bertoni)

Traditionally, Stevia can be propagated using seed or stem cutting. However, the plant seeds show a very low germination percentage [23,24,25] due to inbreeding depression, which causes the production of sterile seeds [26]. Propagation using the seed also causes great variability in stevioside level and composition [27]. Vegetative propagation is limited to a low number of individuals that may be obtained from a single plant and successfully adapted to the soil [28,29]. For commercial purposes, when large-scale propagation is necessary, the conventional method of production is not adequate to fulfil the required demand. The tissue culture is the only alternative method for the mass propagation of *S. rebaudiana* [3]. In recent years, plant tissue culture techniques have become of major industrial importance in the area of plant propagation. Apart from their use as a tool for mass plant propagation, plant tissue culture techniques have also found application in the area of plant conservation, disease elimination, plant improvement and secondary metabolite production [30].

## 3. *Stevia* Tissue Culture Techniques

Various kinds of plant tissue culture techniques such as *in vitro* propagation, genetic engineering, suspension cultures, callus culture, adventitious root culture and elicitation have been developed for mass propagation, biomass and secondary metabolite production of *S. rebaudiana* [31]. The earliest study of Stevia tissue culture found in the literature is by Handro et al. [32]. The authors established that discs from leaves and internodal segments are able to produce vigorous calluses when cultured on synthetic media containing auxin (indole-3-acetic acid—IAA or 2,4-dichlorophenoxyacetic acid—2,4-D) and cytokinin (6-benzyl aminopurine—BAP, kinetin—Kn or zeatin—Z). The best results have been obtained on media with 2,4-D (800 μg/L) and one cytokinin (BAP, Kn or Z, 200 μg/L). In the same research, the addition of adenine was also tested, without any apparent effects. Yang and Chang [33] reported the induction of multiple shoot formation on excised leaflets with a minimum of callus formation *in vitro* on MS media supplemented with 2–10 mg/L BAP with the following root induction on MS medium without hormones. Since then, many researchers have investigated the role of nutrient media, plant growth regulators, different additives and types of explants on the growth, development and steviol glycoside content of Stevia [14,34,35,36,37,38,39,40]. Nowadays, various protocols for its mass propagation *in vitro* have been established. Singh et al. [41] conducted a detailed review of the established protocols for Stevia micropropagation. Generally, *in vitro* regeneration can be achieved by somatic embryogenesis and organogenesis from different explants by manipulating the ratio of cytokinin to auxin in the nutrient medium [42,43]. Bespalhok-Filho and Hattori [14] obtained somatic embryogenesis from floret explants of *S. rebaudiana* cultured on the Murashige and Skoog (MS) [44] medium supplemented with 2,4-D (9.05 and 18.10 µM) and Kn (0 to 9.29 µM). On 9.05 µM of the 2,4-D supplemented medium, maximum embryogenic callus formation occurred without Kn. On 18.10 µM of the 2,4-D supplemented medium, the best treatment was with 2.32 µM Kn. The embryogenic callus started at the base of the corolla and ovary. The organogenesis of Stevia can be achieved from callus [32,45] and suspension [46] cultures, shoot tip [36], axillary bud [47], nodal [34] and leaf [33,46,48] explants, anthers [49] and flowers [50].

## 4. Callus and Suspension Cultures

The callus is referred to as a mass of nondifferentiated cells. A callus culture was obtained by excising an explant from the parent plant and placing it onto a nutrient medium supplemented with an appropriate combination of phytohormones [38,51]. Leaf discs and nodal explants of Stevia are able to produce callus when cultured on MS media supplemented with 2.0 mg/L BAP and 1.0 mg/L α-naphthalene acetic acid (NAA) [52]. Golkar et al. [40] obtained optimum callus growth on the MS medium containing 1 mg/L NAA + 0.5 mg/L BAP. Das et al. [38] reported that Kn in combination with NAA and 2,4-D exhibits better results for callus initiation, whereas the combined application of BAP and NAA in a half-strength MS medium was optimal in maintaining callus. Khalil et al. [53] reported the successful callus-mediated propagation of *S. rebaudiana* from leaf explants. The authors achieved the best callus induction (84.6%) on the MS medium augmented with BAP (2.0 mg/L) and 2,4-D (2.0 mg/L). The highest number of shoots per explant was obtained on a medium containing BAP (1.5 mg/L) and gibberellic acid (GA3; 0.5 mg/L). Suspension cultures are composed of isolated cells and cellular aggregates. Ferreira and Handro [46] described a method for the production, maintenance and plant regeneration of *S. rebaudiana* (Bert.) from cell suspension cultures. These cultures were obtained after 20–30 days by using friable callus as the initial inoculum in liquid media with BAP (0.5 mg/L) + 2,4-D (1.0 mg/L) and periodic filtering (100–500 μm sieves) with a 6–7-day interval between subcultures. Cultures derived from actively growing calli have been primarily diploid (2n = 22), whereas those derived from senescent calli have shown a wide variation in chromosome number (55–200). Stock cell suspensions that had been maintained for 3 years were plated on the basal Linsmaier and Skoog (LS) [54] agar medium with BAP (0.5 mg/L) + 2,4D (0.5 mg/L) to form callus. Calli originating from predominantly 2n cell suspensions, when transferred to a medium with Kn (2.0 mg/L) + NAA (0.02 mg/L), were able to form buds. Shoot elongation and further rooting of isolated shoots were better on an LS medium devoid of growth regulators. Variation in rooting capacity, plant vigor, morphological character and chromosome number was found amongst regenerated plants. Mathur and Shekhawat [55] (2013) established cell suspension cultures of *S. rebaudiana* as a strategy to obtain an *in vitro* stevioside producing cell line. The optimal biomass growth was obtained in a full-strength MS liquid basal medium augmented with 2,4-D (0.27 μM), BAP (0.27 μM) and ascorbic acid (0.06 μM). Callus tissues can sustain the power of cell division for a longer period and are thus suitable for the production of desirable bioactive compounds, germplasm conservation, gene manipulation and genetic engineering [31].

## 5. Direct Organogenesis

Yang et al. [34] (1981) obtained the highest axillary shoot proliferation by culturing nodal explants of *S. rebaudiana* in an MS medium supplemented with BAP (2 mg/L), Kn (10 mg/L) and N6-(2-isopentenyl)-adenine (iP; 10 mg/L). Hwang [56] reported the maximum induction of adventitious shoots from nodal explants on an MS medium supplemented with 2 mg/L IAA and 0.5 mg/L Kn. Ahmed et al. [7] obtained the maximum axillary shoot proliferation from nodal explants cultured on the MS medium with 1.5 mg/L BAP + 0.5 mg/L Kn. Thiyagarajan and Venkatachalam [57] cultivated Stevia nodal explants on the MS basal medium fortified with different concentrations of BAP (0.5–3.0 mg/L) and Kn (0.5–3.0 mg/L) individually for shoot bud induction. The maximum number of shoots (123 shoots/explant) was noted on the MS medium supplemented with 1.0 mg/L BAP after three subcultures on the same media composition. Khan et al. [58] proposed a protocol for the *in vitro* multiplication of *S. rebaudiana* from nodal explants cultivated on the MS medium fortified with 2 mg/L BAP, 1 mg/L NAA and 5 mg/L urea. Tamura et al. [36] established clonal propagation of the plant species by culturing stem tips with a few leaf primordia on the LS agar medium [54] supplemented with a high concentration (10 mg/L) of Kn. The number of shoots yielded from a single shoot tip after 80 days varied from 50 to 100. Shoot primordia were induced from shoot tips on the Gamborg B5 [59] medium containing BAP and NAA [60]. Stevia shoot tip, nodal and leaf explants can regenerate shoots when cultured on an MS medium supplemented with BAP and IAA [37]. Das et al. [38] reported that shoot explants placed on MS media supplemented with Kn (2 mg/L) promoted optimal *in vitro* micropropagation of *S. rebaudiana*. In a study performed by Röck-Okuyucu et al. [39], the effect of different plant growth regulators (PGRs) (BAP, Kn, thidiazuron—TDZ, IAA and NAA) on growth and steviol glycoside content was determined. The authors conclude that a PGR-free medium provides the development of healthy plants with the simultaneous formation of shoots and roots. High-performance liquid chromatography (HPLC) showed higher stevioside and rebaudioside A content in the leaves of Stevia grown on a PGR-free medium compared with the PGR(s)-containing media. Akbari et al. [61] established a positive effect of the addition of inorganic nitrogen sources, i.e., NH_4_NO_3_ (1650 mg/L) and KNO_3_ (950 mg/L), to the MS medium on some morphological traits of *S. rebaudiana* Bertoni derived from axillary nodes as explants. Moreover, the presence of nitrogen sources increases both the shooting and rooting of the plant. Rooting of the *in vitro* derived shoots could be achieved following subculture onto an auxin-containing medium [37]. Tamura et al. [36] used a medium containing NAA (0.1 mg/L) for root formation. A 96% frequency of rooting was achieved after cultivating shoot tips (>2 cm) on a half-strength MS medium augmented with 0.4 mg/L NAA [57]. Khan et al. [58] reported root induction on MS media with NAA (4 mg/L). The rooted plantlets could be successfully transferred into pots containing sand and soil in a ratio of 1:2 and subsequently cultivated in the greenhouse [57]. 

## 6. Genetic Variability

A major problem associated with *in vitro* culture is the possible occurrence of somaclonal variation among the subclones of parental lines [62] due to chromosomal rearrangements, DNA methylations and gene mutation [63,64]. The propagation of plants from callus tissue may bring about frequent genetic variability in the regenerated plants [36,58]. In general, plant populations produced by direct organogenesis from shoot meristems, nodal and leaf explants have been reported to be genetically uniform [58,60,65,66,67,68]. Furthermore, the clonal plants showed significantly smaller variations than the sexually propagated plants; they were almost as homogeneous as the plants propagated by cuttings [66]. Randomly amplified polymorphic DNA (RAPD) analysis of Stevia plants obtained by indirect organogenesis from leaf explants indicated that some of the regenerated plants were 100% similar to the mother plant and some were 71, 57 or 14% similar [69]. In another study, inter simple sequence repeat (ISSR) analysis of Stevia plantlets obtained from callus culture showed genetic variation during the period of culturing, whereas those from nodal segments did not [68]. Soliman et al. [70] used fourteen different ISSR primers to analyze the genetic stability of eight subcultures of Stevia plantlets obtained through multiple shoot regeneration from nodal explants and mother plants. Out of all the initially tested primers, four ISSR primers generated clear, distinct and reproducible bands. All ISSR profiles from the first to fifth subcultures of micropropagated plants have been monomorphic and similar to mother plants, while low variation was induced in the next subculture [70]. In a study conducted by Modi et al. [71], RAPD marker analysis of plants raised *in vitro* from axillary nodes did not show any somaclonal variation. Ramírez-Mosqueda et al. [29] detected 89.6% genetic similarity between *S. rebaudiana* plantlets micropropagated in temporary immersion systems. All these results suggest that clonal propagation via stem-tip culture is an effective method of obtaining a population of uniform plants [67].

## 7. Elicitation

Elicitation is a common treatment used in *in vitro* cultures for obtaining secondary metabolites by using different elicitors [31,72]. A variety of microbial, physical and chemical factors may be considered as elicitors. Some of these elicitors act as signalling molecules, which produce engineered stress conditions in plant cells and initiate the production of reactive oxygen species (ROS), which directly or indirectly enhances the accumulation of secondary metabolites [31]. Several research articles on the positive effects of different elicitors on steviol glycosides biosynthesis in *in vitro*-cultivated *S. rebaudiana* are available in the literature [31]. 

### 7.1. Physical Elicitors

#### 7.1.1. Light

Light is one of the major elicitors that alters the morphogenetic and biochemical responses of plants [73]. Although *S. rebaudiana* is a short-day plant, it was reported that a long-day (16 h) photoperiod induces vegetative growth, thereby significantly increasing leaf biomass and SGs content [8]. In addition to photoperiod, light intensity is also an important factor affecting plant growth and secondary metabolite production. Nakonechnaya et al. [74] established that an LED (light-emitting diode) light with an intensity of 75 and 230 μmol/(m^2^s) provided maximal values of morphometric and mesostructural parameters of Stevia plantlets raised *in vitro*. Several articles regarding the effect of different light spectra on *in vitro* cultivation of plant species are available in the literature. Ahmad et al. [73] investigated the effect of various spectral lights on biomass accumulation and secondary metabolite production in the callus culture of *S. rebaudiana*. Maximum callogenesis (92.73%) was induced by white light followed by yellow (88.34%), blue (76.4%), green (75.12%) and red (64.34%) lights. Callus fresh and dry weights decreased in the order of white-, red-, yellow-, green- and blue-light treatments. Blue light induced the highest phenol and flavonoid content and total antioxidant capacity, while treatment with red and green lights led to the highest 2,2-diphenyl-1-picryl hydrazyl (DPPH) radical scavenging activity [73]. Another study performed with *S. rebaudiana* adventitious root cultures showed that violet light had a stimulating effect on biomass accumulation, while red light exhibited an inhibitory one. As in the above-mentioned study, the maximum accumulation of phenolic compounds was observed after treatment with blue light [75]. The red–blue spectrum treatment had an inhibitory effect on the shoot growth and led to the formation of shortened internodes as compared with the control (fluorescent white lamps). However, root formation increased under these conditions. Five days after returning the shoots to the control treatment, elongation of the newly formed internodes was observed. In response to monochromatic red LED light, shoots with elongated internodes were formed, while under blue LED light, more compact microshoots were observed. However, the multiplication rate was the same after both treatments. As regards the green LED and white fluorescent (control) lights, there have been no significant differences in the shoot height and multiplication coefficient [76].

#### 7.1.2. Temperature

As an abiotic stress factor, temperature strongly influences plants’ growth and secondary metabolism accumulation [77]. Tissue cultures are extremely sensitive to variations in temperatures. The optimal temperatures for plant growth *in vitro* are species specific and usually higher than those observed for the growth of intact plants [78]. The literature data on the influence of temperature on growth and secondary metabolite production in Stevia cultivated *in vitro* are scarce. Mubarak et al. [79] studied the effect of various degrees of temperature (20, 25 and 30 °C) on the *in vitro* growth of the plant, as the highest number of shoots per explant was observed at 25 °C. Yang et al. [80] performed a pot study of *S. rebaudiana* for the evaluation of the influence of low (15 °C) and high (35 °C) temperatures on the transcript levels of fifteen SGs biosynthesis pathway genes. All the genes were transcribed maximally at 25 °C, while both low and high temperatures restrained their transcription.

### 7.2. Chemical Elicitors

Summarized data about the effect of certain elicitors on Stevia growth and secondary metabolite production are given in Table 1.

#### 7.2.1. Plant Growth Regulators

Salicylic acid (SA), methyl jasmonate (Me-JA) and gibberellic acid enhanced the stevioside content in *S. rebaudiana* grown *in vitro* [40,81,82,83,84] (Table 1). Golkar et al. [40] evaluated the effect of different concentrations of salicylic acid (SA) (0.25, 0.5 and 0.75 mg/L) on callus growth and SGs production in the callus culture of Stevia. The addition of SA at a concentration of 0.75 mg/L resulted in the highest level of callus growth rate, callus diameter and relative callus fresh weight. The addition of 0.25 mg/L of SA to the MS medium led to the production of the highest amount of rebaudioside A (3.40 mg/g dry weight callus). Adventitious roots cultured in media supplemented with gibberellic acid (2 mg/L) accumulated SGs in high concentrations [85].

#### 7.2.2. Complex Organic Extracts

Positive effects of certain growth additives such as alginate (ALG), casein hydrolysate (CH), coconut water (CW), malt extract (ME), yeast extract (YE) and chitosan (CHI) on plant growth and secondary metabolite production in *S. rebaudiana* cultured *in vitro* have been reported [82,86] (Table 1). Casein hydrolysate is a complex organic extract and can be a source of calcium, phosphate, several microelements, vitamins and, most importantly, a mixture of up to 18 amino acids [87]. The addition of casein hydrolysate to the woody plant medium (WPM) enhanced stevioside and rebaudioside A content in Stevia [82]. The stevioside content increased 8.38-fold (13.05 mg/g DW) with the treatment of 0.5 g/L CH compared to the control. Although a subsequent decrease in stevioside production was observed with concentrations of 1.0 and 2.0 g/L, both provided higher metabolite content than the control plants [82]. Casein hydrolysate was found to promote the accumulation of stevioside and rebaudioside A in the callus culture of *S. rebaudiana* [88]. The molecular structures of the active ingredients of YE are unknown, but it is known that they can elicit plant defense responses [89,90]. ALG is a polysaccharide present in the cell walls of brown algae. Bayraktar et al. [82] reported an increase in stevioside production from 1.56 mg/g dry weight (DW) to 14.69 and 14.54 mg/g DW in the *in vitro* Stevia plantlets exposed to 0.5 g/L ALG and 2.0 g/L YE, respectively. CW contains growth hormones, and its addition to the nutrient media improved Stevia shoot growth. The optimal concentration in which the maximum shoot length and the number of shoots were observed was 15%, whereas higher concentrations (20%) led to the formation of callus at the cut ends with the following decrease in the shoot multiplication rate [86]. ME is a source of carbohydrates, auxins and gibberellins. Lower concentrations of ME (0.025%, 0.05%) induced Stevia shoot bud initiation and multiplication, whereas higher doses (0.075%, 0.1%) reduced these parameters [86]. CHI is a linear polysaccharide known to induce an increase in the lignin biosynthesis and cell wall lignification of plants, resulting in better shoot growth and an increase in weight [82]. It is the main structural component of the cell wall of plant pathogen fungi, which mimics its effects and activates the biosynthesis of defense-related secondary metabolites in plants [91,92,93,94]. SGs are similar to other secondary plant metabolites in that they behave defensively by feeding deterrents or antimicrobial agents against specific herbivores, pests or pathogens [95,96,97]. The stevioside content was seen to increase in the shoots of *S. rebaudiana* grown *in vitro* in the presence of 50 and 100 μM CHI in the nutrient media, reaching 7.02 mg/g DW (4.51-fold higher than the control plants) at 100 μM [82]. 

**Table 1:** Effect of certain substances on *S. rebaudiana* Bertoni growth and accumulation of bioactive compounds.

Type of Elicitors Used	Effects	References
Salicylic acid	Promotion of callus growth and accumulation of rebaudioside A	Golkar et al., 2019 [40]
	Improvement of stevioside and rebaudioside A production and KA13H, UGT74G1 genes expression	Tahmasi et al., 2017 [81]
Methyl jasmonate	Enhancement of stevioside production in shoot cultures	Bayraktar et al., 2018 [83]
Gibberellic acid	Optimization of biomass production from adventitious root culture and accumulation of polyphenolics and SGs	Ahmad et al., 2020a [85]
	Promotive role in enhancing stevioside content in calluses	Hendawey and Abo El Fadl, 2014 [84]
Casein hydrolysate	An increase in stevioside and rebaudioside A content in callus culture	Hsing et al., 1983 [88]
	Enhancement of *in vitro* shoot regeneration and multiplication	Sridhar et al., 2014 [86]
Chitosan	Acceleration of shoot production and biomass accumulation	Bayraktar et al., 2016; Bayraktar et al., 2018 [82,83]
Alginate	High stevioside accumulation	Bayraktar et al., 2016 [82]
Yeast extract	Improvement of stevioside production	Bayraktar et al., 2016 [82]
Coconut water	Enhancement of shoot growth and development	Sridhar et al., 2014 [86]
Malt extract	Induction of shoots and multiplication	Sridhar et al., 2014 [86]
Proline	Induction of callus growth and stevioside accumulation in callus culture and decrease in malondialdehyde content	Hendawey and Abo El Fadl, 2014 [84]
	Improvement of biomass yield and SGs production in the callus and suspension cultures	Gupta et al., 2015 [98]
Glutamine	Induction of greenish, healthy nodular calli with embryogenic potential.	Das and Mandal, 2010 [99]
	Increase in shoot biomass and chlorophyll content	Thilakavathy and Jagadeesan, 2017 [100]
	Effect on UGT74G1 and UGT76G1 gene expression and steviol glycosides accumulation	Esmaeili et al., 2018 [101]
Creatine lysinate	Beneficial effects on growth parameters and phenol and flavonoid accumulation in *in vitro* cultivated plants	Miladinova-Georgieva et al., 2022 [102]
Glucose	Enhancement of stevioside content in callus cultures	Hendawey and Abo El Fadl, 2014 [84]
Polyethylene glycol	Improvement of biomass yield and SGs production in the callus and suspension cultures	Gupta et al., 2015 [98]
NaCl and Na_2_CO_3_	Reduction in the growth and development of callus and suspension cultures; Increase in the concentration of SGs	Gupta et al., 2014 [103]
NaCl	Increase of antioxidant capacity, hydroxycinnamic acid and total soluble sugar content in *in vitro* plantlets and up-regulation of several genes (CMS, CMK, HDR and UGT76G1) encoding key enzymes of the SGs biosynthetic pathways	Lucho et al., 2019 [104]

#### 7.2.3. Amino Acids

Glutamic acid and proline play a promotive role in enhancing callus growth and stevioside content in Stevia callus culture and this was associated with a clear decrease in malondialdehyde content, which is used as a biomarker to measure oxidative stress [84]. Enhanced production of steviol glycosides (SGs) was observed in the callus and suspension culture of *S. rebaudiana* treated with proline [98]. Das and Mandal [99] found that the addition of glutamine (50 mg/L) to the nutrient medium produced greenish, healthy nodular calli with more embryogenic potential and less necrotic lesion in comparison with only the PGR-supplemented basal medium. In the same study, the effect of tryptophan (50 and 100 mg/L) was also investigated, but no satisfactory effect was observed at all. Thilakavathy and Jagadeesan [100] reported that the total biomass and chlorophyll content of *S. rebaudiana* increased by adding 100 mg/L glutamine in the culture medium. Furthermore, the presence of glutamine in the medium favored an increase in the shoot bud number per explant. Esmaeili et al. [101] have investigated the effect of different concentrations of glutamine (10, 20, 30 and 40 g/L) on the expression of UGT74G1 and UGT76G1 genes (genes that have important roles in the synthesis of stevioside and rebaudioside A) and stevioside and rebaudioside A accumulation in the leaves of Stevia under *in vitro* conditions. The highest level of expression for UGT74G1 was observed in plants grown on MS media without glutamine, while for UGT76G1, the addition of glutamine at concentrations of 2, 3 and 4% has a stimulatory effect. The highest amount of stevioside was accumulated in plants that were under the 3% glutamine treatment, while the highest rebaudioside A accumulation was observed under the 2% glutamine treatment. The lowest accumulation of stevioside and rebaudioside A was observed in plants grown on MS medium without glutamine.

The addition of creatine lysinate to the nutrient medium has a beneficial effect on the growth parameters and accumulation of phenols and flavonoids in *in vitro*-cultivated *S. rebaudiana* Bertoni [102].

### 7.3. Other Organic Components

There are previous studies on the promotive role of glucose and 2-acetooxybenzoic acid on callus growth and stevioside content in Stevia callus culture, and this effect has a been associated with a decrease in the malondialdehyde content [84]. Another study presented a positive effect of polyethylene glycol (PEG) on steviol glycoside (SG) production in the callus and suspension culture of *S. rebaudiana* [98].

#### Sodium Compounds

Stevia plants growing *in vitro* have also been studied for their adaptability to certain abiotic stress factors such as salinity or water stress [9]. The stress conditions created by sodium compounds (NaCl and Na_2_CO_3_) inhibited the growth of callus and suspension cultures, but significantly enhanced the accumulation of SGs [103]. The biomass growth decreased from 113.60 mg in the control to 93.66 mg (in 0.10% NaCl treatment) and 99.93 mg (in 0.025% Na_2_CO_3_ treatment). In callus, the quantity of SGs increased over five-fold from 0.27 (control) to 1.43 and 1.57% with 0.10% NaCl and 0.025% Na_2_CO_3_, respectively. In the case of the suspension culture, the same concentrations of NaCl and Na_2_CO_3_ increased the SG content from 1.36 (control) to 2.61 and 5.14%, respectively, on the 10th day. Treatment with NaCl increased the antioxidant capacity (according to the ferric reducing/antioxidant power—FRAP assay), hydroxycinnamic acid and total soluble sugar content of *S. rebaudiana in vitro* plantlets and led to the up-regulation of several genes (CMS, CMK, HDR and UGT76G1) encoding key enzymes of the steviol glycoside biosynthetic pathways [104].

## 8. Nanoparticles

Numerous reports indicate positive aspects of nanoparticle application in plant tissue culture [105]. Because of their size (between 1 and 100 nm), nanoparticles (NPs) exhibit unique physicochemical properties such as a large surface-area-to-volume ratio, the ability to engineer electron exchanges, special electronic and optical attributes and surface reactive capability [106,107,108]. Nanoparticles have been widely used to improve seed germination, plant growth and yield, secondary metabolites production and plant protection [105,106,108,109,110,111]. Furthermore, they find applications as disinfectants, nutrients and elicitors, as tools for genetic transformation, callus induction, organogenesis and somatic embryogenesis [112,113]. Nanoparticles exert their action through various pathways, such as their effects on ethylene, enzymes, nitrogen metabolism and light harvesting complex II (LHC II). No toxicity on plant growth was detected if the permissive concentration was considered [110] (Table 2).

A few studies on the effect of different nanoparticles on the growth and secondary metabolite production of *Stevia in vitro* have been reported. Hendawey et al. [10] found appropriate concentrations of Fe_3_O_4_NPs, CuONPs and SiO_2_NPs that positively affect antioxidant enzymes (SOD and CAT) activity and stevioside production in the *Stevia* callus culture. Ahmad et al. [121] investigated the influence of ZnO and CuONPs on *in vitro* root formation, non-enzymatic antioxidant activities and the production of SGs in *in vitro*-cultivated *Stevia* plants. The highest percentage of rooting, the greatest content of SGs (rebaudioside A and stevioside) and the highest total flavonoid content, total phenolic content and 2,2-diphenyl-1-picryl hydrazyl-free radical scavenging activity were observed at 2 mg/L of ZnO and 20 mg/L of CuO NPs. Both ZnO and CuO NPs at 200 mg/L and 2000 mg/L concentrations induced adverse effects on plant biomass, antioxidant activities and SG content. Hence, the biochemical and morphophysiological responses of Candyleaf were elicited as a defense against CuO and ZnO NPs applied under the threshold limit [121]. Javed et al. [116,117] investigated the effects of different concentrations (0, 0.1, 1.0, 10, 100 or 1000 mg/L) of ZnO (34 nm in size) and CuO (with an average size of 47 nm) nanoparticles on growth parameters, steviol glycosides (rebaudioside A and stevioside) production and antioxidant activities in *S. rebaudiana* Bertoni grown *in vitro*. The highest percentage of shoot formation, as well as a significant enhancement of steviol glycosides in micropropagated shoots, was observed at 1 mg/L of ZnO and 10 mg/L CuO NPs. The oxidative stress produced by CuO and ZnO nanoparticles on *S. rebaudiana* was affirmed by increased DPPH scavenging activity, total antioxidant capacity, total reducing power, total flavonoid content and total phenolic content, with increasing oxidative pressure and generation of ROS. However, the antioxidant activities, formation of secondary metabolites and physiological parameters showed a sudden decline after crossing the threshold of a 1 mg/L concentration of ZnONPs and falling to a minimum at 1000 mg/L, elucidating the maximum phytotoxic effect of ZnONPs at this concentration [117]. The highest amount of total phenolic content (TPC) and total flavonoid content (TFC), as well as the total antioxidant capacity (TAC) and DPPH free radical scavenging activity in callus culture of *Stevia,* have been obtained at 100 mg/L of ZnONPs, whereas TPC, TAC, TRP (total reducing power) and DPPH free radical scavenging activity achieved the highest values at a 10 mg/L concentration of CuONPs. The highest TRP in the context of ZnO and the highest TFC regarding CuO have been achieved at 50 and 100 mg/L, respectively [123]. Ghazal et al. [118] reported that the addition of bimetallic alloys of gold and copper nanoparticles (AuCu NPs) to the liquid MS medium containing 0.5 mg/L NAA has a positive effect on the production of biomass and secondary metabolites in adventitious root cultures of *S. rebaudiana.* The maximum total phenolics content and total flavonoid content, as well as the maximum DPPH free radical scavenging activity, were displayed using AuCu NPs at a ratio of 1:3. A study by Khan et al. [122] indicated that lower concentrations of FeNPs (45 µg/L) had a positive influence on the morphological growth parameters of the plant species. At a higher dose (90 and 135 µg/L), FeNPs in culture media were found to be detrimental to growth characteristics and development. Furthermore, the stress caused by FeNPs at 135 µg/L in cultures produced higher levels of total phenolic content, total flavonoid content and antioxidant activity. In addition, plants grown in the presence of FeNPs at 90 µg/L resulted in higher enzymatic antioxidant activities (SOD, POD, CAT and APX). Furthermore, exposure to a low dose of FeNPs (45 µg/L) exhibited the maximum amount of SGs (stevioside and rebaudioside A) as compared to high doses. The nodal explants of *Stevia* were cultured *in vitro* on an MS medium where the normal source of Zn was replaced with different concentrations of ZnNPs (50, 100, 200, 400 and 1000 mg/L) [114]. The ZnNPs showed potential toxicity to the plant, which was reflected in both the morphological data and the production of ROS scavenging enzymes under different treatment conditions. The 200 mg/L concentration of ZnNP showed lower toxicity symptoms. The phytotoxicity severity increased at a concentration of 400 mg/L and above with the highest toxicity at a 1000 mg/L concentration. The production of stevioside was significantly reduced when exposed to ZnNP in a concentration-dependent manner. The study showed the concentration-dependent phytotoxic effect of ZnNP on the physiology and stevioside production in *Stevia* [114]. A similar study was performed by Desai et al. [115] with MgNPs. Nodal explants of *Stevia* were cultured on the MS medium where the normal source of Mg^+^ was replaced with MgNPs (at 125, 250, 500, 750 and 1000 mg/L). An MS medium devoid of MgSO4.7H_2_O was used as a negative control. The authors revealed that Mg NPs are able to replace their bulk counterparts and effectively eliminate Mg^+^ deficiency in plants. The MgNPs improved the plant morphology (leaf length, leaf width, shoot length, the number of nodes per explant, internodal space and number of shoots per explant) as compared to the negative control. The activities of ROS scavenging enzymes (CAT, GPOX and SOD) increased in a concentration-dependent manner upon MgNPs treatment, as these activities were lower compared to plants grown on Mg^+^-deficient media and higher than those reported with positive control (MS basal medium). An Mg^+^ deficiency leads to a drastic reduction in stevioside content, whereas the application of MgNPs enhances it in a concentration-dependent manner, with the maximum stevioside content at 1000 mg/L of MgNPs [115]. In *S. rebaudiana*, high doses of AgNPs were reported to accumulate in plant cells and tissues [124]. AgNPs were shown to be present in epidermal stem cells, within vascular bundles and in intermembrane spaces [119]. In leaves, they were observed in ribs and stomata. An increase in Ag content in tissues was observed as AgNPs concentrations in the culture medium increased [119]. Castro-González et al. [119] conducted a study to determine the response of *S. rebaudiana* B. to different concentrations of silver nanoparticles (0, 12.5, 25, 50,100 and 200 mg/L AgNPs) added to the nutrient medium. The lowest AgNP concentrations (12.5 mg/L) promoted the greatest shoot production and length per explant. An increase in chlorophyll a, b and total contents was observed after all treatments with AgNPs. The dry matter gradually increases from 25 mg/L of AgNPs, obtaining the highest values with 100 and 200 mg/L AgNPs concentrations. Golkar et al. [40] evaluated the effect of different concentrations of silver nanoparticles (AgNPs) (15, 30, 45 and 60 mg/L) on callus growth and SGs production in the callus culture of *Stevia*. The addition of AgNPs at a concentration of 45 mg/L led to the highest amount of stevioside (32.34 mg/g dry weight callus). Ramezani et al. [120] studied the effect of AgNPs at various concentrations (0, 10, 20 and 40 mM) on the transcription of key gene levels in the biosynthesis of rebaudioside and stevioside glycosides, including UGT85C2 (UDP-glycosyltransferases), KAH (Kaurenoic acid-13 hydroxylase), UGT74G1 and UGT76G1 in *Stevia* using real-time PCR assays. UGT85C2 demonstrated the highest transcriptional level changes in plants treated with silver nanoparticles. The plants treated with silver nanoparticles at concentrations of 10 and 20 mM showed a lower gene expression than the control plant, but the plant treated with silver nanoparticles at a 40 mM concentration showed significantly higher gene expression than the control samples. The results suggested that treatment with AgNPs at 40 mM leads to similar positive effects on the transcription of all genes. HPLC results also revealed that the plants treated with 40 mM AgNPs contain a higher glycosides content compared to the control sample. Thus, this experiment suggests that AgNPs can act as a strong amplifier of the transcriptional trigger for steviol glycoside biosynthesis pathway genes, which have the potential to control the production of steviol glycosides positively [120]. 

Sichanova et al. [125] evaluated the impact of aminoacid nanofibers carriers of 1% and 2% colloidal silver (NF-1% Ag and NF-2% Ag) on *in vitro* cultivation and steviol glycoside content of *S. rebaudiana* Bertoni. The research showed a hormetic effect of the nanofibers—at low concentrations, from 1 to 50 mg/L, stimulation of plant growth was observed, while at high concentrations (100 mg/L), they possessed a harmful effect. The highest stevioside content was measured in plants treated with 100 mg/L NF-1% Ag.

## 9. Effect of Elicitors on Antioxidant Activity in *S. rebaudiana*

*S. rebaudiana* exhibits strong antioxidant potential due to the presence of various enzymatic and non-enzymatic molecules with antioxidant functions. The major enzymatic components involved in protecting plant cells from oxidative stress are superoxide dismutase (SOD), catalase (CAT), guaiacol peroxidase (GPX), glutathione reductase (GR) and ascorbate peroxidase (APX) [126]. The non-enzymatic antioxidants in which *Stevia* is rich are phenolics, flavonoids, diterpene glycosides, tannins and anthocyanins [127]. The first evaluation of the antioxidant potential of *Stevia in vitro* was performed by Sadhana et al. [128] who worked with leaf and callus extracts. The total phenolic content was 25.18 mg/g DW (dry weight) for leaves and 35.86 mg/g DW for callus. The flavonoid content was 21.73 and 31.99 mg/g in the leaf and callus, respectively. Total antioxidant activity measured by the ferric-reducing/antioxidant power (FRAP) assay was equivalent to 25.70 and 35.16 mg/g DW ascorbic acid in water and methanolic extract of the leaves, respectively. In the case of callus, it was 25.11 mg/g DW for the water extract and 32.32 mg/g DW for the methanolic extract. The percent inhibition of the DPPH radical with the water extract of leaves and callus was 39.86% and 55.42%, respectively, whereas it was 33.17% and 56.82% for the methanolic extract of leaves and callus, respectively. Comparing the antioxidant capacity of the leaf extract of *Stevia* grown *in vitro* with that from field-adapted ex vitro plants, Zayova et al. [129] established that the latter possess higher amounts of water-soluble antioxidants, phenols and flavonoids, hence higher total antioxidant capacity (expressed as a percentage inhibition of the free radical DPPH) was measured. This can be explained by the greater variety of stress factors to which field-grown plants were exposed in comparison with plants grown under controlled *in vitro* conditions. The antioxidant activity of the *in vitro*-cultivated plants can be influenced by the modulation of culture conditions and medium composition [130]. The amount of proline, an excellent stress indicator, and the activity of antioxidant enzymes SOD, CAT and APX increased in *Stevia* callus under fluoride-induced stress [131]. Radić et al. [130] evaluated the effect of different pH levels (4.6, 5.8 and 7.4) of the culture medium on the accumulation of secondary metabolites and free radical scavenging activity in *S. rebaudiana*. The maximum accumulation of total phenols and total flavonoids, as well as the highest values of DPPH radical inhibition, were recorded in the leaves and roots of the plants grown on a medium with a pH value of 4.6.

A study conducted by Miladinova-Georgieva et al. [102] shows a favorable effect of creatine and creatine lysinate on the physiological processes of *in vitro*-cultivated *S. rebaudiana* Bertoni. The activities of antioxidant enzymes CAT, APX and GPX decreased after treatment with most of the studied concentrations of these amino acids, while SOD activity and the content of lipid-soluble antioxidants increased gradually with increasing concentrations of creatine and creatine lysinate (1, 5 and 10 mg/L). The number of water-soluble antioxidants increased at the highest concentration of the amino acids (10 mg/L). The results obtained by the FRAP method led authors to assume that lysine in a higher concentration suppresses the effect of creatine—creatine increases the antioxidant activity measured by the FRAP method and lysine returns it closer to the control. The DPPH assay showed that creatine alone and in combination with lysine as a salt did not alter the overall antioxidant status of the plant.

## 10. Conclusions

Plant tissue culture techniques are increasingly used for the mass propagation of *S. rebaudiana* Bert. Cooperative applications of both traditional and biotechnological tools enable the generation of plants with desired agronomic traits such as disease resistance, improved biomass and high glycoside content. In recent years, many efforts have been made to clarify the role of nanoparticles as a novel elicitor for plants’ primary and secondary metabolism. There are different and often conflicting reports on the effect of NPs on the growth and development of various plant species. Many questions concerning the fate and toxicity of NPs in plant cells remain unsolved.

## Figures and Tables

**Table 2:** Types and optimal concentrations of nanoparticles used for enhancement of the growth and secondary metabolite production of Stevia grown *in vitro*.

Nanoparticles	Optimal Concentration	References
Fe_3_O_4_ NPs CuO NPs SiO_2_ NPs		Hendawey et al., 2015 [10]
Zn NPs	200 mg/L	Desai et al., 2015 [114]
Mg NPs	1000 mg/L	Desai et al., 2017 [115]
CuO NPs	10 mg/L	Javed et al., 2017a [116]
ZnO NPs	1 mg/L	Javed et al., 2017b [117]
AuCu NPs		Ghazal et al., 2018 [118]
Ag NPs	45 mg/L	Golkar et al., 2019 [40]
Ag NPs	100 mg/L; 200 mg/L	Castro-González et al., 2019 [119]
Ag NPs	40 mM	Ramezani et al., 2020 [120]
ZnO CuONPs	2 mg/L 20 mg/L	Ahmad et al., 2020b [121]
Fe NPs	45 μg/L	Khan et al., 2020 [122]

## Data Availability

Not applicable.

## Abbreviations

2:4-D—2,4-dichlorophenoxyacetic acid; ALG—alginate; APX—ascorbate peroxidase; BAP—6-benzyl aminopurine; CAT—catalase; CH—casein hydrolysate; CHI—chitosan; CW—cococnut water; DPPH—2,2-diphenyl-1-picryl hydrazyl; DW—dry weight; FW—fresh weight; FRAP—ferric reducing/antioxidant power; GA3—gibberellic acid; GPX—guaiacol peroxidase; GR—glutathione reductase; IAA—indole-3-acetic acid; HPLC—high-performance liquid chromatography; iP—N6-(2-isopentenyl)-adenine; ISSR—inter simple sequence repeat; Kn—kinetin; LED—light-emitting diode; LS—Linsmaier and Skoog; ME—malt extract; Me-JA—methyl jasmonate; MS- Murashige and Skoog; NAA—α-naphthalene acetic acid; NPs—nanoparticles; PGRs—plant growth regulators; RAPD—Randomly amplified polymorphic DNA; ROS—reactive oxygen species; SA—salicylic acid; SGs-steviol glycosides; SOD—superoxide dismutase; TAC—total antioxidant capacity; TFC—total flavonoid content; TPC—total phenolic content; TRP—total reducing power; WPM—woody plant medium; YE—yeast extract; Z—zeatin.
